# Prevalence and determinants of undernutrition among under-five children residing in urban slums and rural area, Maharashtra, India: a community-based cross-sectional study

**DOI:** 10.1186/s12889-020-09642-0

**Published:** 2020-10-16

**Authors:** Sujata Murarkar, Jayashree Gothankar, Prakash Doke, Prasad Pore, Sanjay Lalwani, Girish Dhumale, Sanjay Quraishi, Reshma Patil, Vivek Waghachavare, Randhir Dhobale, Kirti Rasote, Sonali Palkar, Nandini Malshe

**Affiliations:** 1 Department of Community Medicine, Bharati Vidyapeeth Deemed to be University Medical College, Off Pune Satara Road, Pune, 411043 India; 2 Department of Pediatrics, Bharati Vidyapeeth Deemed to be University Medical College, Pune, India; 3 Department of Community Medicine, Bharati Vidyapeeth Deemed to be University Medical College, Sangli, India; 4 Department of Community Medicine, Symbiosis Medical College for women, Pune, Maharashtra India

**Keywords:** Undernutrition, Under-five children, Rural area, Urban slum

## Abstract

**Background:**

Undernutrition among under five children in India is a major public health problem. Despite India’s growth in the economy, the child mortality rate due to undernutrition is still high in both urban and rural areas. Studies that focus on urban slums are scarce. Hence the present study was carried out to assess the prevalence and determinants of undernutrition in children under five in Maharashtra, India.

**Methods:**

A community-based cross-sectional study was conducted in 16 randomly selected clusters in two districts of Maharashtra state, India. Data were collected through house to house survey by interviewing mothers of under five children. Total 2929 mothers and their 3671 under five children were covered. Multivariate logistic regression analysis was carried out to identify the determinants of child nutritional status seperately in urban and rural areas.

**Results:**

The mean age of the children was 2.38 years (±SD 1.36) and mean age of mothers was 24.25 years (± SD 6.37). Overall prevalence of stunting among children under five was 45.9%, wasting was 17.1 and 35.4% children were underweight. Prevalence of wasting, stunting and underweight were more seen in an urban slum than a rural area. In the rural areas exclusive breast feeding (*p* < 0.001) and acute diarrhea (*p* = 0.001) were associated with wasting, children with birth order 2 or less than 2 were associated with stunting and exclusive breast feeding (*p* < 0.05) and low maternal education were associated with underweight. Whereas in the urban slums exclusive breast feeding (*p* < 0.05) was associated with wasting, sex of the child (*p* < 0.05) and type of family (*p* < 0.05) were associated with stunting,and low income of the family (*p* < 0.05) was associated with underweight.

**Conclusions:**

Factors like sex of the child, birth order,exclusive breast feeding,economic status of the family, type of family,acute diarrhea and maternal education have influence on nutritional status of the child. Improvement of maternal education will improve the nutritional status of the child. Strategies are needed to improve the economic status of the community.

**Trial registration:**

Trial registration number: CTRI/2017/12/010881; Registration date:14/12/2017. Retrospectively registered.

## Background

Undernutrition among under five children in India is a major public health problem [1]. Its prevalence is highest in the world and is almost double that of Sub-Saharan Africa [2, 3].

Out of the total world’s undernourished children, 80 % lives in 20 countries. In India almost 60 million children are underweight [4] .

UNICEF in the year 2006 reported the causes of childhood malnutrition as insufficient diet, frequent infections, poor breastfeeding practices, delayed introduction of complementary foods and inadequate protein in the diet. Other factors that influence food intake include health status, food taboos, growth and personal choice related to diet. Malnutrition can also develop due to neglect, abnormal mealtimes, insufficient quantities of food and insufficient parental knowledge [5].

Every year International Food Policy and Research Institute (IFPRI) publishes the Global Hunger Index (GHI). According to the report for 2019, India is ranked 102 out of a total of 119 countries [6]. Prevalence of undernutrition among under five children according to the National family health survey 4 (NFHS 4) in India shows that 35.7% under five children were underweight, 38.4% were stunted and 21% were wasted [7]. From National family health survey 1 to National family health survey 4, the prevalence of undernutrition has not declined as desired. According to the Comprehensive National Nutrition Survey report (2016–2018),35% of Indian children aged 0–4 years were stunted, 17% were wasted and 33% were underweight [8]. Malnutrition in the form of undernutrition, namely underweight, stunting and wasting has been coined as the “silent emergency” by the United Nations children’s fund [9–11].

The government of India has strongly committed to achieving the 2030 Sustainable Development Goals (SDGs). End hunger, achieve food security, and improved nutrition and promote sustainable agriculture, all these nutrition-related factors are included in sustainable development goals (SDGs) [12]. If undernutrition is not effectively reduced, the country will not meet its SDG target of child mortality reduction [8].

In developing countries, the nutritional status of children depends on socioeconomic status, awareness of diseases such as diarrhea and acute respiratory tract infection, educational status of mother and availability of safe drinking water [13]. Undernourished children are prone to infections. Statistically underweight children succumb to diseases such as diarrhea, measles, and malaria and lower respiratory tract infections. Undernutrition in young children has long-term negative effects on physical and cognitive development [14]. A global review on child stunting and economic outcomes revealed a 1 cm increase in height was associated with a 4% increase in wages for men and a 6% increase in wages for women. Investing in the reduction of child malnutrition is paramount for human and economic development [8]. Data from six longitudinal studies on the association between anthropometric status and mortality of children aged 6–59 months revealed a strong association between the severity of weight-for-age deficits and mortality rates. Indeed, out of the 11.6 million deaths among under-five children in 1995 in developing countries, it has been estimated that 6.3 million or 54% of young child mortality were associated with malnutrition [15].

Despite India’s growth in the economy, the child mortality rate due to undernutrition is still high in both urban and rural areas. Hence assessment of nutritional status among children is critical in framing health policies [16].

The majority of earlier studies on undernutrition were carried out either in the rural area or in the urban area, while few earlier studies focus only on chronic undernutrition i.e. stunting. To bridge this gap,the present study was conducted to assess the prevalence of undernutrition in children under five and its determinants in rural and urban areas of Maharashtra, India.

## Methods

This study was a part of the baseline survey conducted in 16 clusters located in two districts of western Maharashtra, India, during cluster randomized control trial (CRCT) among children below 5 years of the rural area and urban slums. The trial was registered with clinical trial registry of India (CTRI).

### Study design

A community-based cross-sectional study.

### Study setting

The current study was carried out in rural areas and urban slums in two districts in Maharashtra state, India. Composite health index (Sangli = 0.66 & Pune = 0.65) of these two districts are similar as well as they are similar in health indicators [17]. The rural and urban areas have revenue villages and slums, respectively. These slums or revenue villages are referred to as clusters. There are total 45 clusters (Pune district = 20, Sangli district = 25). On average there are 250 under five children per cluster and a total population of 1, 89,504 [18].

### Study duration

The main CRCT was conducted between 15th December 2015 to 14th March 2018. Benchmark data i.e. data of this study was collected between 15 Feb 2016 to 14 May 2016.

### Sample size

Out of 45 clusters, 8 clusters each in intervention and comparison arm were selected. In selected clusters, all the under five children were included in the survey. However, we had tested the adequacy of the sample size. Considering the National prevalence of underweight (35.7%), stunting (38.4%) and wasting (21%) sample size was independently calculated for underweight, stunting and wasting [7]. With a 95% confidence interval and allowable difference of 10% of prevalence, sample size 1504 was calculated by using the following formula.

$$ \mathrm{Sample}\ \mathrm{size}={{\mathrm{Z}}^2}_{\alpha}\mathrm{p}\ \left(1-\mathrm{p}\right)/{\mathrm{d}}^2 $$

With cluster effect 2, the sample size came around 3008. By considering a 10% loss to follow up we need to study 3309 children, but we had included 3671 children which is an adequate sample size.

### Study participants

All children below 5 years with their mothers in the selected areas.

### Research tool

The questionnaire was first prepared in English**.** It was validated by a panel of experts and then translated into the local language(Marathi) and retranslated in English (Additional file 1). Inimitable identification code was given to each house, the mother of a child below 5 years and the child.

### Data collection

Eight field supervisors (FS) appointed for the project collected data from house to house survey with the help of Accredited Social Health Activists (ASHAs) in all areas except Pune urban slum where anganwadi workers (AWWs) cooperation was sought. Designated site investigators (SI) monitored and supervised the activities of each field supervisor. These site investigators were faculty of the two medical colleges. In the selected clusters, all families residing for more than 6 months were included in the study. Households that were found to be locked during two consecutive visits were not included in the study. According to standard guidelines, weight measurements were recorded to the nearest 100 g using the standard weighing machine and the height of the children was recorded to the nearest 0.1 cm using a measuring tape. The exact age of the child was computed from the child’s date of birth. When data on the exact date of birth was not available, the age as told by the mother was used, to the nearest month.

#### Definitions used in the present study

1. Type of family: It was divided into two groups; the nuclear family consists of the husband, wife and unmarried children staying together, the joint family included all other families including three-generation family as well as extended family. Households, where its occupants were not blood- related i.e. household occupied by nonblood related members, were not considered as family and not included in the study.
2. Socio-economic status: In Maharashtra state, there are three types of ration cards, based on the income of the family. Accordingly, families were classified under three categories depending on income considering the color of the card as a proxy for income. The yellow ration card represents less than or up to Rs. 15,000 (205.97 USD), an orange ration card represents between Rs. 15,000 (205.97 USD) to Rs 100,000 (1373.15 USD) and white ration card represents more than Rs.100, 000 (1373.15 USD) income per annum [19].
3. Educational status including literacy status of mothers: A person who could not read and write was labeled as illiterate [20]. According to years of schooling educational status was classified as education up-to 6th standard,7th to 10th standard, 12th standard/Diploma, Graduate and above.
4. Birth order: Birth order of the child was considered as told by the mother.
5. Immunization status (for children between 12 and 23 months):
  - Fully immunized Children- Those who have received all vaccines recommended in National immunization schedule in infancy i.e. BCG, OPV zero dose and Hepatitis B at birth, DPT1 and OPV1 at 6 weeks, DPT2 and OPV 2 at 10 weeks, DPT 3 and OPV3 at 14 weeks, Measles and vitamin A at 9 months.
  - Non immunized children -Those who have not received a single vaccine.
  - Partially immunized- All other children were considered as partially immunized
  - Children with immunization card were considered for the analysis.
6. Exclusive breastfeeding: Child fed only breast milk except taking vitamins, mineral supplements, or medicines until 6 months of age was considered as exclusive breastfeeding [21].
7. Wasting in children: According to WHO standards child with weight for height z -score below − 2 Standard Deviations (SD) of the median of a reference standard [22].
8. Stunting in children: According to WHO standards child with length/height for age z -score below − 2 Standard Deviations (SD) of the median of a reference standard [22].
9. Underweight children: According to WHO standards child with weight for age z -score below − 2 Standard Deviations (SD) of the median of a reference standard [22].
10. CRCT - Cluster randomized control trial is a comparative study in which the units randomised are pre-existing (natural or self-selected) groups whose members have an identifiable feature in common, and in which outcomes are measured in all, or a representative sample of the individual members of the groups [23].
11. CTRI- Clinical Trial Registry of India is a online record system meant for registering all clinical trials conducted in India [24].
12. ASHA- Accredited social health activist works at village level under National health mission programme of India. She creates awareness on health in the community [25].
13. AWW- Under the Integrated child development services (ICDS) scheme anganwadi workers(part time workers) are appointed to render health services in the community [25].
14. SPSS-Statistical package for social sciences is a software package used for statistical analysis.

### Data processing

All filled forms were entered into the software database. To check completeness and accuracy in the form, critical fields in the tool were identified. Along with critical fields, noncritical fields were also monitored. For critical data discrepancies up to 0.1% and and for non-critical data upto 1% were considered acceptable. For discrepancies related to data entry alternate forms were physically cross-checked. Complete cases were analyzed and missing data were not included because of low occurrence. The data were analyzed after cleaning the data and final report was prepared.

### Data analysis

Data were analyzed using statistical package for social sciences (SPSS) (version 20). Variables adjusted during analysis were as follows- for maternal education illiterate and all categories less than 6th standard were merged together as ≤6th standard whereas all the categories above 6th standard were merged together as>6th standard. During analysis of type of family, families other than joint and nuclear were merged in joint family type. While analyzing exclusive breast feeding < 6 months and 6 months these to categories were merged together. The findings were reported in terms of frequency and percentage for qualitative variables and quantitative variable findings were shown by descriptive statistics. Multivariate logistic regression analysis was carried out to identify the determinants of child nutritional status seperately in urban and rural areas. Independent variables like sex of the child, type of family, income of the family, birth order, exclusive breastfeeding, immunization status, ARI, diarrhea, maternal age, maternal education were taken into consideration and the dependent variables were wasting,stunting and underweight in rural area and urban slum. As for immunization of children we had considered all doses within 1 year, the sample size for that variable got reduced. So due to the minimum sample size of this variable and multi-collinearity in covariables, this variable got removed from the multivariate logistic regression analysis. Results were reported by 95%confidence interval with a 5% level of significance. *P*-value of less than 0.05 (*p* < 0.05) was considered significant.

## Results

In the present study, 2929 mothers with 3671 under five children were enumerated [18]. Out of the total population children below 5 years constituted 7.5%. There were 1834 under-five children in urban slum and 1837 in the rural area. There were 1939 boys and 1732 girls in the study area. The mean age of the children was 2.38 years (±SD 1.36). Out of these 3671 under five children, 752 were infants. The mean birth weight of these infants was 2.6 kg (±SD 0.61). Almost 80.1% women were in the age group between 20 and 29 years. The mean age of mothers was 24.25 years (±SD6.37). About 56.55% of mothers had education up-to high school.

Exclusive breastfeeding (EBF) was given to 46% of the children. It was seen higher in children from a rural area (78%) than in children from an urban slum (15%) (*P* < 0.05).

There were a total of 768 children in the age group of 12 to 23 months but only 639 children possessed immunization card. Out of these 639 children, 94.6% were fully immunized. It was higher in rural clusters (97%) than urban clusters (91%) (P < 0.05). For 129 children, immunization information was missing as their status could not be confirmed by immunization card. We have studied the association of factors affecting undernutrition like the gender of the child, type of family, the income of the family, birth order, exclusive breastfeeding, immunization status, ARI, diarrhea, maternal age and maternal education.

Table 1 shows the types of under-nutrition in rural area and urban slums. Out of total 3671 under five children data of 3.5% of children were missing on the day of the survey, hence the total does not match. Overall, 17.1% under five children were wasted, 45.9% were stunted and 35.4% were underweight. It was observed that the prevalence of all three types of under-nutrition, wasting (OR = 0.836), stunting (OR = 0.737) and underweight (OR = 0.886) was less in rural areas. Stunting was the most common form of under-nutrition observed.

**Table 1:** Prevalence of undernutrition among under five children in the rural area and urban slum

Status of Undernutrition		Area		Total (%)	Odds ratio(95%CI)
		Rural area (%)	Urban slum (%)		
Wasting (*n* = 3530)	Yes	282(15.9)	322(18.4)	604(17.1)	0.836 (0.701–0.996)
	No	1497(84.1)	1429(81.6)	2926(82.9)	
Stunting (*n* = 3563)	Yes	751(42.1)	885(49.7)	1636(45.9)	0.737 (0.646–0.842)
	No	1031(57.9)	896(50.3)	1927(54.1)	
Underweight (*n* = 3557)	Yes	610(34.0)	649(36.8)	1259(35.4)	0.886 (0.772–1.016)
	No	1183(66.0)	1115(63.2)	2298(64.6)	

Exclusive breastfeeding in both rural area (Adj OR = 0.35,*p* < 0.001) and urban slum (Adj OR = 0.47,*p* < 0.05) and diarrhea in the rural area (Adj OR = 0.11,*p* = 0.001) are the determinants of wasting among under-five children (Table 2). In the computation of adjusted odds ratio of all the variables in table number 2, 3 and 4, following reference categories were considered – male sex of the child,exclusive breast feeding > 6 months, nuclear type of family,income of family≤1373.15 USD,birth order > 2,diarrhea -yes, ARI-yes,maternal age ≤ 20 and maternal education ≤6th standard.

**Table 2:** Determinants of wasting among under five children in the rural area and urban slum

Determinants of wasting		Rural area					Urban slum				
		Wasting		Total	Adj OR (CI)	p-value	Wasting		Total	Adj OR (CI)	p-value
		Yes	No				Yes	No			
Sex of child	Male	177	761	938	0.63(0.36–1.09)	0.096	187	733	920	1.16(0.70–1.94)	0.567
	Female	105	736	841			135	696	831		
Exclusive BF till 6 months of age	No	49	130	179	0.35(0.19–0.63)	< 0.001	65	141	206	0.47(0.27–0.83)	0.009
	Yes	27	164	191			22	102	124		
Type of Family	Nuclear	114	681	795	0.81(0.46–1.42)	0.461	129	549	678	0.98(0.55–1.76)	0.952
	Joint	167	808	975			193	873	1066		
Income of Family	≤ 1373.15 USD	135	679	814	1.39(0.80–2.41)	0.245	247	994	1241	0.98(0.53–1.78)	0.935
	> 1373.15 USD	146	810	956			75	428	503		
Birth order	> 2	11	34	45	0.86(0.38–1.82)	0.644	12	52	64	1.82(0.89–3.72)	0.101
	≤2	64	258	322			75	191	266		
Diarrhea	Yes	21	68	89	0.11(0.03–0.41)	0.001	9	31	40	0.01(0.00- > 10)	0.999
	No	261	1429	1690			313	1398	1711		
ARI	Yes	141	824	965	0.99(0.56–1.74)	0.97	173	657	830	0.83(0.49–1.39)	0.472
	No	141	673	814			149	772	921		
Maternal age	≤20	21	139	160	1.83(0.82–4.06)	0.138	38	109	147	1.39(0.69–2.80)	0.361
	> 20	254	1313	1567			272	1277	1549		
Maternal Education	≤6th standard	42	182	224	0.68(0.30–1.55)	0.358	56	240	296	0.73(0.36–1.49)	0.383
	>6th standard	238	1285	1523			255	1162	1417		

It was observed that stunting among under-five children was significantly associated with the sex of the child in the urban slum(Adj. OR = 1.77,*p* < 0.05), birth order in the rural area (Adj OR = 2.11,*p* < 0.05) and type of family in the urban slum (Adj OR = 0.56,*p* < 0.05) (Table 3).

**Table 3:** Determinants of stunting among under five children in the rural area and urban slum

Determinants of stunting		Rural area					Urban slum				
		Stunting		Total	Adj OR (CI)	p-value	Stunting		Total	Adj OR (CI)	p-value
		Yes	No				Yes	No			
Sex of child	Boy	404	535	939	1.45(0.95–2.23)	0.087	462	471	933	1.77(1.12–2.87)	0.015
	Girl	347	496	843			423	425	848		
Exclusive BF till 6 months of age	No	88	92	180	1.31(0.84–2.05)	0.232	115	94	209	1.45(0.90–2.34)	0.13
	Yes	103	88	191			78	49	127		
Type of Family	Nuclear	341	455	796	1.33(0.85–2.07)	0.213	375	320	695	0.56(0.32–0.95)	0.033
	Joint	409	568	977			507	572	1079		
Income of Family	≤ 1373.15 USD	362	454	816	0.80(0.52–1.24)	0.316	655	612	1267	0.96(0.56–1.63)	0.877
	> 1373.15 USD	388	569	957			227	280	507		
Birth order	> 2	16	29	45	2.11(1.06–4.19)	0.033	34	32	66	1.44(0.80–2.58)	0.221
	≤2	173	150	323			159	111	270		
Diarrhea	Yes	35	55	90	0.64(0.17–2.42)	0.514	18	22	40	0.01(0.00- > 10)	0.999
	No	716	976	1692			867	874	1741		
ARI	Yes	418	548	966	1.21(0.78–1.89)	0.394	399	437	836	1.33(0.84–2.12)	0.223
	No	333	483	816			486	459	945		
Maternal age	≤20	84	76	160	0.59(0.33–1.04)	0.067	80	68	148	0.85(0.46–1.58)	0.601
	> 20	650	920	1570			783	794	1577		
Maternal Education	≤6th standard	112	113	225	0.82(0.41–1.65)	0.578	170	136	306	0.74(0.38–1.45)	0.381
	>6th standard	628	897	1525			700	736	1436		

Important factors influencing underweight among children were exclusive breastfeeding up to 6 months in rural area (Adj OR = 0.50,*p* < 0.05), income of family in urban slum (Adj OR = 2.16,*p* < 0.05) and maternal education in rural area (Adj OR = 0.44,*p* < 0.05) (Table 4).

**Table 4:** Determinants of underweight among under five children in the rural area and urban slum

Determinants of underweight		Rural area					Urban slum				
		Underweight		Total	Adj OR (CI)	p-value	Underweight		Total	Adj OR (CI)	p-value
		Yes	No				Yes	No			
Sex of child	Male	355	596	951	0.74(0.46–1.18)	0.201	367	562	929	0.68(0.43–1.08)	0.106
	Female	255	587	842			282	553	835		
Exclusive BF till 6 months of age	No	65	115	180	0.50(0.30–0.82)	0.006	83	129	212	1.06(0.66–1.71)	0.815
	Yes	48	145	193			52	72	124		
Type of Family	Nuclear	275	522	797	1.06(0.65–1.73)	0.814	247	436	683	0.75(0.44–1.28)	0.293
	Joint	333	654	987			400	674	1074		
Income of Family	≤ 1373.15 USD	274	547	821	1.31(0.81–2.11)	0.273	457	794	1251	2.16(1.27–3.65)	0.004
	> 1373.15 USD	334	629	963			190	316	506		
Birth order	> 2	10	35	45	1.69(0.78–3.68)	0.187	24	40	64	1.07(0.59–1.95)	0.826
	≤2	101	224	325			111	162	273		
Diarrhea	Yes	37	54	91	0.29(0.08–1.07)	0.064	16	25	41	0.01(0.00- > 10)	0.999
	No	573	1129	1702			633	1090	1723		
ARI	Yes	333	640	973	0.92(0.56–1.50	0.724	311	526	837	0.97(0.61–1.54)	0.885
	No	277	543	820			338	589	927		
Maternal age	≤20	54	106	160	0.88(0.48–1.62)	0.69	51	99	150	1.02(0.55–1.90)	0.941
	> 20	544	1036	1580			578	981	1559		
Maternal education	≤6th standard	96	129	225	0.44(0.21–0.89)	0.023	108	190	298	1.55(0.77–3.10)	0.215
	>6th standard	508	1027	1535			526	902	1428		

Thus multiple logistic regression analysis confirmed the factors such as sex of the child, exclusive breastfeeding, type of family, income of the family, birth order of the child, diarrhea (past 1 month) and maternal education as the determinants for undernutrition among under-five children.

## Discussion

The current study was a large comparative study as compared to most of the studies in India among under five children of urban slum and rural area [1–3]. This study is unique in providing information about undernutrition and it’s determinants at the community level. The data were collected by qualified and trained field supervisors and the quality checks for data collection was monitored by the site investigators from public health, community medicine and pediatrics specialty.

It was observed that overall prevalence of stunting among children under five was 45.9%, wasting was 17.1 and 35.4% children were underweight. Similar statistics emerge from several studies in India [8, 26, 27]. High prevalence of stunting (89.6%) and underweight (73.2%) were reported in a study among under-five children in Uttar Pradesh, India [28]. Whereas another study had revealed a low prevalence of both underweight (19.9%) and stunting (17.1%) and reversed the ranking [3].

Wasting may result from inadequate food intake or a recent episode of illness-causing weight loss. The highest prevalence of wasting is in South Asia, where approximately one in six children (16%) are moderately or severely wasted. The burden of wasting is highest in India, which has more than 25 million (20%) wasted children. This exceeds the combined burden of the next nine high-burden countries [29]. Overall, 17% of Indian children age 0–4 years were wasted [8], which is as per the present study finding.

Stunting reflects chronic undernutrition and hence UNICEF is also focusing on stunting among under five children [30]. In the present study, we found that stunting was more prevalent in the urban slum (49.7%) as compared to rural area (42.1) whereas according to Comprehensive National nutrition survey report (2016–2018) higher prevalence of stunting in under-fives was found in rural areas (37%) as compared to urban areas (27%) [8]. We feel that the availability of food grains are more or less uniform throughout the year in an urban slum. In rural area seasonal availability of food grains increases. There is a tendency to purchase only locally produced and available seasonal grains, vegetables, and fruits which are comparatively cheaper in rural areas. Hence stunting is more prevalent in the urban area.

Many socio-demographic characteristics of the child and family are associated with the presence of undernutrition. They include the gender of the child, birth weight, birth order, number of siblings, exclusive breastfeeding, immunization status, mother’s education and occupation, family income, mother’s knowledge about the timing of weaning and diet, etc. Out of these factors’ majority and particularly most critical we assessed in the present study.

It was seen that exclusive breastfeeding up to 6 months gives protection against wasting to children both from a rural area (Adj OR = 0.35, *p* < 0.001) and urban slum (Adj OR = 0.47, *p* < 0.05). Exclusive breastfeeding up to 6 months acts as a protective factor against infection because it is rich in anti-infective factors that prevent respiratory infections and diarrheal diseases. It enhances the immunity of the child. However, it should be accompanied by timely weaning. Continuing exclusive breastfeeding beyond 6 months implies the child is getting inadequate nutrition and become malnourished.

Childhood infections like diarrhea and acute respiratory tract infection are important causes of malnutrition among under-five children in developing countries. As these are acute episodes, it results in immediate weight loss. In the present study prevalence of wasting was found higher in under-five children with acute diarrhea in the rural area (Adj OR = 0.11, *P* = 0.001), a similar finding was supported by other studies [31–34].

Birth order has always been an important determinant of undernutrition. As compared to children with birth order 2 or more than 2, children with birth order less than two were more likely to be stunted in the rural area (Adj OR = 2.11, *p* < 0.05). This finding was opposite to findings from other studies in India [11, 26, 27, 35]. It may be because in Maharashtra teenage marriages are common [7]. It triggers the link of early childbearing, low birthweight babies which results in developing long term undernutrition of the child. It was observed that the prevalence of stunting was more among boys as compared to girls (Adj OR = 1.77, *P* < 0.05) in the urban slum. This is in line with other studies [1, 14, 36, 37]. Although the exact reason was not known, it may be due to the well-known fact that the male child is more affected by environmental stress than a female child. Contrary to this finding study from South West Rajasthan reported that stunting was 1.48 times more in females than in male children (OR = 1.48; Cl = 1.00–2.47) [38].

Family plays an important role in health and disease. It was seen that the joint family gives protection against stunting to under-five children of the urban slum (Adj OR = 0.56, *p* < 0.05). This emphasizes the importance of a joint family in society. The Indians understand the importance of a joint family system since time immemorial. Sharing resources and responsibilities amongst family members can help parents reduce the economical and physical stress. Children also get more attention in the joint family. Association between the type of family and undernutrition was not found significant by other studies [38, 39].

As per the recommendation of global public health, for achieving optimum growth, development, and health a child should be breastfed exclusively during the first 6 months of life. To evolve as a healthy individual, the infant should be continued with adequate and appropriate safe complimentary food along with breast milk up to 2 years of age or beyond [5]. In the present study also, similar finding was observed i.e. exclusive breastfeeding up to 6 months acts as a protective factor against underweight among under five children from a rural area (Adj OR = 0.50, *p* < 0.05). This finding was supported by other studies [9, 40].

It is well known that socioeconomic status is one of the important determinants of the wellbeing of children and health [41]. Lower the socioeconomic status higher is the risk of undernutrition. A supportive study done in India and Africa reveals that families with low economic status have a significant association with undernutrition [6]. With the improvement in socioeconomic status undernutrition proportionately declines [42]. It was observed that the low income of the family had resulted in underweight among children from an urban slum (Adj OR = 2.16,*p* < 0.05). A similar finding was also revealed by a study in urban slums of Delhi [43].

Another important factor affecting underweight was maternal education. It was observed that low maternal education was a risk factor for undernutrition among under five children of rural areas (Adj OR = 0.44, *p* < 0.05). Undernutrition decreases with an increase in the educational qualification of the mothers. This was in line with other studies [9, 44, 45]. Mother is a universally first caregiver for the child and hence mother’s education matters. Educated mothers are more aware of the health services available and the acceptance to utilize the same is better among them. Mother is also the first teacher of the child and hence mother and child are treated as one unit. Educated girls marry at slightly higher age comparative to less educated girls and accordingly late childbearing and have a fewer number of children.

## Conclusions

Study results indicate that under nutrition is still an important health concern among under five children. Undernutrition in the form of underweight and stunting is more prevalent than wasting in the urban slum and rural area. Factors like sex of the child, birth order,exclusive breast feeding,economic status of the family, type of family,acute diarrhea and maternal education have influence on nutritional status of the child. Improvement of maternal education will improve the nutritional status of the child. Strategies are needed to improve the economic status of the community.

### Limitations

As immunization status was based on immunization card, children who had received some immunizations but lost their immunization card would be misclassified.

We have not taken dietary survey of under five children.

## Supplementary information

**Additional file 1.** Study questionnaire. Study questionnaire used for data collection.

## Data Availability

Corresponding author Dr. Jayashree Gothankar has the datasets analysed during the current study. Data can be accessed by requesting her.

## Abbreviations

CRCT: Cluster Randomized Control Trial
CTRI: Clinical Trial Registry of India
ARI: Acute respiratory infections
EBF: Exclusive breastfeeding
ASHA: Accredited social health activist
AWW: Anganwadi worker
FS: Field supervisor
SI: Site Investigator
SPSS: Statistical package for social sciences
OR: Odds ratio
CI: Confidence interval
UNICEF: United Nations Children’s Fund
WHO: World Health Organization
