# Supplementary material for: Prevalence and determinants of undernutrition among under-five children residing in urban slums and rural area, Maharashtra, India: a community-based cross-sectional study
Source: BMC Public Health. 2020 Oct 16;20:1559. doi: 10.1186/s12889-020-09642-0 (PMC7565769; doi:10.1186/s12889-020-09642-0)
Supplement: Supplementary file 1 — Additional file 1. Study questionnaire. Study questionnaire used for data collection. [file 12889_2020_9642_MOESM1_ESM.pdf]

Department of Community Medicine, BVDUMC, Pune

BASELINE FORM*

** To be filled from all households from the selected clusters.*

Household code (7 Digits)			
Date of interview	DD / MM / YYYY		
Name of Interviewer	Last Name	First Name	Middle Name
Name of Respondent	Last Name	First Name	Middle Name
Name of Family Head	Last Name	First Name	Middle Name

❖ *INSTRUCTIONS: CIRCLE THE CORRECT RESPONSE.*

Education of Family Head	1. Illiterate 2.Can read & write (but not went to school)<br>3. 1-3 pass 4.4-6 pass 5. 7-9 pass 6. SSC<br>7. HSC 8. Diploma 9. Graduate 10. PG/ PhD
Address with Landmark	

Land Line No.		Mobile No.	

1.	For how long you are staying here?	_______________ years
2.	Type of Family	1. Joint 2.Nuclear 3.Other
3.	Which colored ration card do you have?	1. White 2. Orange 3. Yellow<br>4. No Card
4.	Religion	1. Hindu 2. Muslim 3. Christian<br>4.Buddhist 5. Sindhi 6. Sikh<br>7. Parsi 8. Others__________________
5.	Caste	1. SC 2. ST 3. NT/VJNT<br>4. OBC 5. Open 6. Others<br>____
6.	Total family members in the house	
7.	Are there children under the age of five years in the family?	1. Yes 2. No
	❖ **If answer to Q. No. 7 is 'No' then go to Q. No. 9**	

BASELINE FORM*

			Name	Date of Birth	Age (yrs&mnt)
8.	If yes, write details	1		DD/MM/YYYY	______ ______
		2		DD/MM/YYYY	______ ______
		3		DD/MM/YYYY	______ ______
		4		DD/MM/YYYY	______ ______
		5		DD/MM/YYYY	______ ______
		6		DD/MM/YYYY	______ ______

9.	How many married women in age group of 15 – 45 yrs. are in the house?	0 1 2 3 4 5
10.	How many pregnant women are there in the house?	0 1 2 3 4 5
	**If answer to Q. No. 10 is 'zero' then go to Q. No. 12**	
11.	If pregnant mother is in the house/family write months of gestation	1st pregnant woman ________ months.<br>2nd pregnant woman ________ months.<br>3rd pregnant woman ________ months.
12.	How many children under five years of age died in the last year (15/02/2015 to 14/02/2016) in your house/family	0 1 2 3

❖ *If Answer to Q. No. 12 is "zero", or there is death then inform Sub-investigator and Then go to Q. No. 17.*

13.	Is death certificate available? (Interviewer to look for the death certificate)	1st death 1. Yes 2.No<br>2nd death 1. Yes 2.No<br>3rd death 1. Yes 2.No

❖ *If Answer to Q. No. 13 is "No", Then go to Q. No. 15.*

			Cause of death
14.	Write cause of death from the death certificate.	1st death	
		2nd death	
		3rd death	

			Symptoms	How many days
15.	Write the symptoms present child before death	1st death		

BASELINE FORM*

		2nd death		
		3rd death		
16.	Was the death due to respiratory disease? (where available cause of death to be confirmed from death certificate or symptoms)	1st death 1. Yes 2.No<br>2nd death 1. Yes 2.No<br>3rd death 1. Yes 2.No		
**Housing and Environment History**				
17.	Total area of house in square feet	______________________sqft. (carpet area)		
18.	Per capita square feet area (Conclusion from Q. No. 17 & 6)			
19.	Overcrowding (Conclusion)			
20.	No. of rooms in house	0 1 2 3 4 5		
21.	Flooring of house	1. Tiled 2. Earthen (covered/ not covered by cow dung) 3. Cementing (koba)		
22.	Ceiling /table fan present in house	0 1 2 3 4 5		
23.	Exhaust fan	1. Present 2. Absent		
24.	Inference: Ventilation			
25.	Type of fuel used inside the house	1. LPG 2. Biomass fuel (cow dung cake/ wood etc.)<br>3. Stove with kerosene 4. Electric shegdi<br>5.Shegdi with coil		
If answer to Q. No. 25 is other than "2" then go to Q. No. 27				
26.	If using biomass fuel; does the family uses smokeless chullah	1. Yes 2. No		
27.	Does anybody smoke in the house	1. Yes 2. No		

❖ *If Answer to Q. No. 27 is "No", Then go to Q. No. 29.*

		Name of the person who smokes	Relation with child #	Type (1.Bidi/2.Cigarette)	Number/ day	Does smoke when inside the home (1.Yes / 2.No)
28.	1.					
	2.					
	3.					

BASELINE FORM*

	# 1-Father, 2-Mother, 3-Brother, 4-Sister, 5-GrandFather, 6-GrandMother, 7-Uncle, 8-Aunty, 9-Others.	
29	Is tobacco is burned at your house for preparation of *Mishri?*	0. No 1. Daily 2. Alternate day<br>3. Twice a week 4. Once a week<br>5. Other _____

FAMILY INFORMATION

30 Information about family members (married daughters staying at some other place should not be included in the list)

Sr. No.	Complete Name	Age	Gender 1. Male/ 2. Female	Education$	Occupation#	Annual Income (Rs.)
1						
2						
3						
4						
5						
6						
7						
8						
9						
10						
31.	Total Annual family income					
32.	Per capita monthly income					

$ 0. Age < 7 years (Not applicable) 1. Illiterate 2.Can read & write (but not went to school) 3. 1-3 pass 4.4-6 pass 5. 7-9 pass 6. SSC 7. HSC 8. Diploma 9. Graduate 10. PG/ PhD

0. Age < 14 years (Not applicable) 1. Profession; 2. Semi-Profession; 3.Service; 4. Skilled Worker; 5. Semi-Skilled Worker; 6. Unskilled worker; 7.Homemaker 8.Unemployed 9. Retired 10. Student

FORM FOR CHILD UNDER 5 YEARS (0 to 4 years364 days)

Household code (7 digit)			
Mother code (8th digit)			
Child Code (9th digit)			
Child Name			
1.	Birth registered? (Ask for the birth certificate)	1. Yes 2. No	
2.	Height of child	___________cms	Weight ___________kgs
3.	Mothers Name		
4.	Is the child registered in Anganwadi?	1. Yes 2. No	
❖ *If Answer to Q. No. 4 is "Yes", then go to Q. No. 6.*			
5	If not Why?		
6.	Does child go to Anganwadi regularly?(> 20 days/month)	1. Yes 2. No	
❖ *If Answer to Q. No. 6 is "Yes", then go to Q. No.8.*			
7.	If no Why?		
8.	Has child suffered from ARI (cough/cold) in last one month from the date of interview?	1. Yes 2. No	
9.	If Yes how many times	1 2 3	
10.	If child had cough/cold was he/she given home remedies	1. Yes 2. No	
❖ *If answer to Q. No. 10 is no, then go to Q. No. 12*			
11.	Which home remedies were given?		
12.	Was your child admitted in hospital for any major illness in the past?	1. Yes 2. No	
❖ *If Answer to Q. No. 12 is "No", then go to Q. No. 14.*			
13.	If yes, which disease?		
14.	Is the child suffering from any illness currently?	1. Yes 2. No	
❖ *If Answer to Q. No. 14 is "No", then go to Q. No. 16.*			

FORM FOR CHILD UNDER 5 YEARS (0 to 4 years364 days)

15.	If yes, which illness?			
16.	How many times did the child suffered from pneumonia in last one year?		0 1 2 3	
	If response to Q. 16 is zero, ask and circle to Yes for the signs and symptoms during any ARI episode.			
17.	a.	**Cough**	1. Yes	2. No
	b.	**Fever Present**	1. Yes	2. No
	c.	**Flaring of nostrils**	1. Yes	2. No
	d.	**Fast breathing**	1. Yes	2. No
	e.	**In-drawing of the chest**	1. Yes	2. No
	f.	Noisy breathing	1. Yes	2. No
	g.	Wheezing sound	1. Yes	2. No
	h.	Cyanosis	1. Yes	2. No
	i.	Was child unconscious during the episode	1. Yes	2. No
	j.	Were there any sluggish movements of the child during the episode	1. Yes	2. No
**CONCLUSION FOR PNEUMONIA**			1. Yes	2. No
**If answer to any one Q. No. 17 a to c is 'Yes' and answer to any one Q. No. 17 d to e is 'Yes' then conclusion is pneumonia.**				
**DETAILS OF PNEUMONIA**				
Ask following details if child has suffered from pneumonia in last one year or conclusion is "Yes"				
**PNEUMONIA EPISODE – 1**				
18.	1. Who told that the child had pneumonia?		1. Doctor 2. Nurse 3. ASHA<br>4. Anganwadi Sevika 5.Others<br>______________ 6. Interviewer's opinion	
	2. Place of treatment		1. Govt. Facility 2.Private<br>3. Others ________________________	
	3. Whether the child was admitted in hospital?		1. Yes 2. No	
	4. if yes duration		___________days	
	5. Duration of treatment		___________days	
	6. Total expenditure of treatment		Rs.______________________	
	Verify the above information based on records			

FORM FOR CHILD UNDER 5 YEARS (0 to 4 years364 days)

	7. Verified?	1. Yes 2. No
**PNEUMONIA EPISODE – 2**		
19.	1. Who told that the child had pneumonia?	1. Doctor 2. Nurse 3. ASHA<br>4. Anganwadi Sevika 5.Others<br>______________ 6. Interviewer's opinion
	2. Place of treatment	1. Govt. Facility 2.Private<br>3. Others ____________________________________
	3. Whether the child was admitted in hospital?	1. Yes 2. No
	4. if yes duration	___________days
	5. Duration of treatment	___________days
	6. Total expenditure of treatment	Rs.______________________________
	Verify the above information based on records	
	7. Verified?	1. Yes 2. No
**Ask following questions about last episode**		
20.	Which transport facility you had used to visit to a doctor/facility?	
	1. Two wheeler	2. Rickshaw
	3. Tempo	4. Bus
	5. Private vehicle	6. Government Ambulance including 108
	7. Private Ambulance	8. Walking
	9. Other	

21.	How much distance you have to travel to take treatment for Pneumonia? And How much time it took?			
	1. Kms		2. Time	_____hours _______min

22.	Did you have to borrow/sale/mortgage of assets for the treatment of the Child?	1. Yes 2. No
23.	Was there any loss of work due to child's pneumonia? (write '0' if no days)	Father ________________days<br>Mother _______________days
24.	Which practices you followed at home when child was having Pneumonia (including home remedies if any)	

FORM FOR CHILD UNDER 5 YEARS (0 to 4 years364 days)		
25.	Had you informed ASHA/AWW/ Health worker about Pneumonia episode?	1. Yes 2. No
26.	Did ASHA/AWW help you during treatment of child?	1. Yes 2. No

ADDITIONAL INFORMATION FORM FOR INFANTS (0-1 year)

Mother code (8th digit)		
Child Code (9th digit)		
Child Name		
1.	Place of birth	1. Gov. Hospital 2. Pvt. Hospital 3. Home 4. While going to Hospital 5. Other __________
2.	Type of delivery	1. Cesarean 2. Normal
3.	Weight at the time of Birth	1.______________kg (from mother statement)<br>2.______________kg (from records)
4.	If place of delivery is home, then who conducted the delivery	1. Doctor 2. Nurse 3. Trained Dai 4. Untrained Dai 5. Other ________________________________
5.	Birth order of this child	1 2 3 4 5
6.	Did the baby cry immediately after birth?	1. Yes 2. No
7.	Did you feed colostrum to the baby?	1. Yes 2. No
	❖ *If Answer to Q. No. 7 is "Yes", then go to Q. No. 9.*	
8.	Why?	
9.	When did you start the breastfeeding after delivery?	0. Within half an hour 1. Within 1 hour 2. Within 2 hours 3. Within 4 hours 4. Within 24 hours 5. On 2nd days 6. On 3rd day 7. After 3 days 8. No breast feeding at all.
	❖ *If Answer to Q. No. 9 is "Yes", then go to Q. No. 10.*	
10.	If option 8 is selected ask WHY?	
11.	What was the first feed to the baby	1. Breast milk (colostrum) 2. Honey 3.

ADDITIONAL INFORMATION FORM FOR INFANTS (0-1 year)

		Sugar water 4. Guti<br>5. Other _______________
12.	For how many months did you give the child exclusive breast milk only?	1. < 6 months 2. Up to 6 months<br>3. > 6 months 4. Not applicable
13.	Weaning (solid food) was started at the age?	1. _________months 2. Not yet given 3. Not applicable
14.	Are you still breastfeeding the child after weaning?	1. Yes 2. No.<br>3. Weaning not yet started
15.	At what age did you stop breastfeeding completely?	1. Months ______<br>2. Not applicable since still breast feeding.

IMMUNIZATION DETAILS (FOR AGE GROUP 12-23 MONTHS)

Mother's code:	
Childs name	
Child's code:	

Confirm all details from immunization card. Don't include pulse polio doses. Write date from card.

Sr. No.	Age	Vaccine	1.Given 2.Not given		Date
1	After birth	BCG	1	2	
		OPV 0 (zero polio)	1	2	
		HBV -0	1	2	
2	6 weeks (1.5 months)	OPV-1	1	2	
		DPT-1	1	2	
		HBV-1	1	2	
		Pentavalent-1	1	2	
		Rotavirus -1	1	2	
		Pneumococcal -1	1	2	
		Hib – 1	1	2	
		IPV	1	2	
3	10 weeks (2.5 months)	OPV-2	1	2	
		DPT-2	1	2	
		HBV-2	1	2	
		Pentavalent-2	1	2	
		Rotavirus -2	1	2	
		Pneumococcal -2	1	2	
		Hib – 2	1	2	
		IPV	1	2	
4	14 weeks (3.5 months)	OPV-3	1	2	
		DPT-3	1	2	
		HBV-3	1	2	
		Pentavalent-3	1	2	
		Rotavirus -3	1	2	
		Pneumococcal -3	1	2	
		Hib – 3	1	2	
		IPV	1	2	
5	9 months	Measles-1	1	2	
		Vitamin A	1	2	
6	16 months	Vitamin A	1	2	
7	16-24 months	DPT –booster	1	2	
		OPV- booster	1	2	
		Measles-2	1	2	
		MMR	1	2	
		Pneumococcal conjugate -booster	1	2	
		Hib booster	1	2	
		Chickenpox	1	2	
		HAV	1	2	
8	24 months	Vitamin A	1	2	
Whether child has received BCG, 3 doses of DPT, OPV, HBV and 1 dose of measles within one year of age			1. Yes	2. No	

KAP of Mothers

Name of Mother	
Mother Code (8th digit)	☐☐☐☐☐☐☐☐

❖ Circle the correct response

1.	Which are the common illnesses in under five children? (multiple response)	1. Fever 2. ARI 3. Pneumonia<br>4. Diarrhea 5. Measles 6. Malnutrition<br>7. Other ______________________________
2.	Can ARI become a serious condition?	1. Yes 2. No
3.	Can ARI be prevented?	1. Yes 2. No
4.	For how many months an infant should be given exclusive breast feeding?	________months
5.	Measles vaccine is given at what age?	
6.	What are the effects of smoking of a family member on child's health?	
7.	Knowledge about hand hygiene	
	a. When to wash your (mother's) hands?	1. ……………………………………………..<br>2. ……………………………………………..<br>3. ……………………………………………..<br>4. ……………………………………………..
	b. What to use for washing hands?	1. Water only 2. Ash<br>3. Soil 4. Soap and water<br>5. Others …………………………………..
8.	Which are the risk factors from the list for pneumonia?(multiple response)	
	1. Low birth weight	2. Prematurity
	3. Hypertension of mother	4. Non-exclusive breast feeding for six months
	5. Nuclear Family	6. Smoking by family member in the house
	7. Malnourishment of child	8. Baby born by LSCS

KAP of Mothers		
	9. Poor hand hygiene in mother	10. Use of chulha or biomass in house
	11. age of mother < 20 years	12. Overcrowding in house
	13. Others	14. Don't Know
9.	What are the symptoms of pneumonia? (multiple response)	
	1. Fever	2. Cold
	3. Cough	4. Difficulty in breathing
	5. Increased rate of breathing	6. Noisy breathing
	7. Flaring of nostrils	8. Chest in drawing
	9. Other	10. Don't know
	If answer to Q. No. 9 is "Don't know" then go to Q. No. 11	
10.	What are the danger signs of Pneumonia from above when you need to show the child to some doctor or health worker? (multiple response)	
	1. Fever	2. Cold
	3. Cough	4. Difficulty in breathing
	5. Increased rate of breathing	6. Noisy breathing
	7. Flaring of nostrils	8. Chest in drawing
	9. Other	10. Don't know
11.	Where will you go first to take treatment if your child gets illness like Pneumonia?	1. Government 2. Private 3.Others
12.	What is/are the reason/s for the choice of the treatment Facility (multiple response)	
	1. Closest Facility	2. Good care offered
	3. Only facility Accessible	4. Financial Treatment Affordability
	5. Short waiting time	6. Personal relation to personnel
	7. High trust in skill of personnel	8. Other
13.	Can Pneumonia cause death?	1. Yes 2. No
14.	Should government think of Pneumonia control activities?	1. Yes 2. No
15.	Is mother's hygiene important?	1. Yes 2. No

KAP of Mothers

16.	If yes, then specify	
17.	Practice about hand hygiene	
	a. When do you wash hands?	1. ……………………………………………..<br>2. ……………………………………………..<br>3. ……………………………………………..<br>4. ……………………………………………..
	b. What material do you use to wash your hands?	1. Water only 2. Ash<br>3. Soil 4. Soap and water<br>5. Others …………………………………..
